# Genome-wide association study reveals novel genetic locus associated with intra-individual variability in response time

**DOI:** 10.1038/s41398-018-0262-z

**Published:** 2018-10-04

**Authors:** Ari Pinar, Ziarih Hawi, Tarrant Cummins, Beth Johnson, Marc Pauper, Janette Tong, Jeggan Tiego, Amy Finlay, Marieke Klein, Barbara Franke, Alex Fornito, Mark A. Bellgrove

**Affiliations:** 1 School of Psychological Sciences and Monash Institute for Cognitive and Clinical Neurosciences (MICCN), Monash University, Melbourne, VIC Australia; 2 Department of Human Genetics, Donders Institute for Brain, Cognition and Behaviour, Radboud University Medical Center, Nijmegen, The Netherlands; 3 Donders Institute for Brain, Cognition and Behaviour, Radboud University Medical Center, Nijmegen, The Netherlands; 4 Department of Psychiatry, Radboud University Medical Center, Nijmegen, The Netherlands

## Abstract

Intra-individual response time variability (IIRTV) is proposed as a viable endophenotype for many psychiatric disorders, particularly attention-deficit hyperactivity disorder (ADHD). Here we assessed whether IIRTV was associated with common DNA variation genome-wide and whether IIRTV mediated the relationship between any associated loci and self-reported ADHD symptoms. A final data set from 857 Australian young adults (489 females and 368 males; *M*_age_ = 22.14 years, SD_age_ = 4.82 years) who completed five response time tasks and self-reported symptoms of ADHD using the Conners’ Adult ADHD Rating Scale was used. Principal components analysis (PCA) on these response time measures (standard deviation of reaction times and the intra-individual coefficient of variation) produced two variability factors (labelled response selection and selective attention). To understand the genetic drivers of IIRTV we performed a genome-wide association analysis (GWAS) on these PCA-derived indices of IIRTV. For the selective attention variability factor, we identified one single-nucleotide polymorphism (SNP) attaining genome-wide significance; rs62182100 in the *HDAC4* gene located on chromosome 2q37. A bootstrapping mediation analysis demonstrated that the selective attention variability factor mediated the relationship between rs62182100 and self-reported ADHD symptoms. Our findings provide the first evidence of a genome-wide significant SNP association with IIRTV and support the potential utility of IIRTV as a valid endophenotype for ADHD symptoms. However, limitations of this study suggest that these observations should be interpreted with caution until replication samples become available.

## Introduction

A hallmark of neurocognitive disturbance in many neurological and psychiatric disorders is an increase in intra-individual variability in response time (IIRTV). This increase has been observed across a range of cognitive tasks and disorders, including stroke^1^, Alzheimer’s disease^2^, schizophrenia^3,4^, major depressive disorder^4^, bipolar disorder^5^, and attention-deficit hyperactivity disorder (ADHD; in children and adults)^6–8^. In ADHD, increased IIRTV has been suggested as a leading endophenotype that is able to index the underlying genetic risk for the disorder^9,10^. Several behavioural genetic studies have revealed the heritable nature of intra-individual variability, driven largely by additive genetic influences^11,12^. Rather than simply representing a non-specific marker of brain dysfunction, IIRTV has been associated with neurobiological networks of attention^13–15^. Indeed, neuroimaging studies in both adolescents and adults suggest that IIRTV is related to task-dependent activations of the anterior cingulate and medial prefrontal cortex, dorsolateral prefrontal cortex, and posterior parietal cortex^13–16^. Collectively, these findings have led to the proposition that IIRTV arises from the involvement of two processing streams; a top-down attentional control process whereby IIRTV results from spontaneous fluctuations in attentional demand, or a bottom-up process wherein IIRTV develops due to disruptions of the “default mode” network of the brain, comprising regions that are typically deactivated during goal-orientated response time (RT) tasks^17,18^.

Despite growing support for both a genetic and neurobiological basis of IIRTV, its specific genetic architecture remains largely unknown. Although a small number of candidate gene studies have identified associations with catecholamine system genes such as *DAT1*, *DRD4*, *SLC6A2* and *ADRA2A* in cohorts of individuals with and without ADHD^19–22^ the additive genetic influences of IIRTV have yet to be explored in a genome-wide manner.

The aim of the present study was to perform a genome-wide association study (GWAS) of IIRTV in a general population sample of young adults. Specifically, we explored IIRTV across a range of neurocognitive measures of RT performance, and conducted principal component analyses (PCAs) to reduce the dimensionality of the RT data into two components, representative of response selection and selective attention, respectively. We then performed a genome-wide association analysis on these PCA-derived indices of IIRTV to identify novel sources of genetic variation contributing to IIRTV. Finally, we explored the association between our PCA-derived indices of IIRTV and self-reported ADHD symptoms, and examined whether the former mediated the relationship between individual genetic variants associated with IIRTV and self-reported ratings of ADHD symptoms. Using this methodology, we identify the first genetic locus associated with IIRTV at a genome-wide level.

## Materials and methods

### Participants

One thousand two hundred and ninety-six control participants with no self-reported personal history of psychiatric or neurological disorders were recruited for the present study. Participants were recruited from Melbourne and Brisbane, Australia. To control for genetic variation amongst ethnic populations all participants were of European ancestry as determined by self-report of the ancestry of all four grandparents^23^. Ethical approvals were granted by both the University of Queensland and Monash University ethics committees. All participants provided informed consent before completing a battery of five cognitive RT tasks and self-reporting ADHD symptoms using the long version of the Conners’ Adult ADHD Rating Scales (CAARS-S:L)^24^. Saliva samples for genetic analysis were obtained from participants using Oragene kits (DNAgenotek, Kanata, Ontario, Canada), and DNA was isolated from those according to standard protocols.

### Stimuli and procedures

**Fig. 1:**
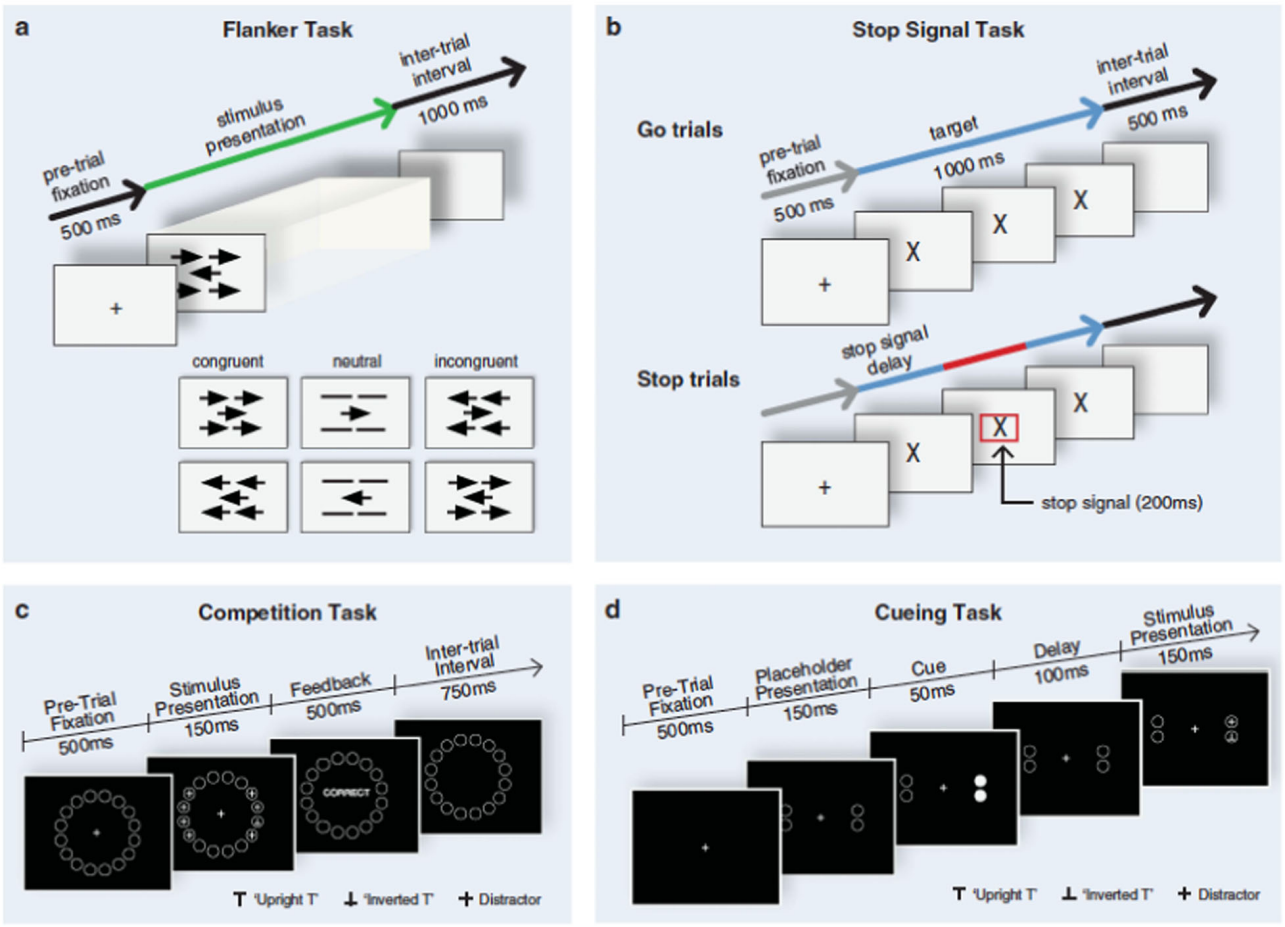
Schematic depictions of the five response time tasks utilised by the present study as previously reported ^22^

**Fig. 2:**
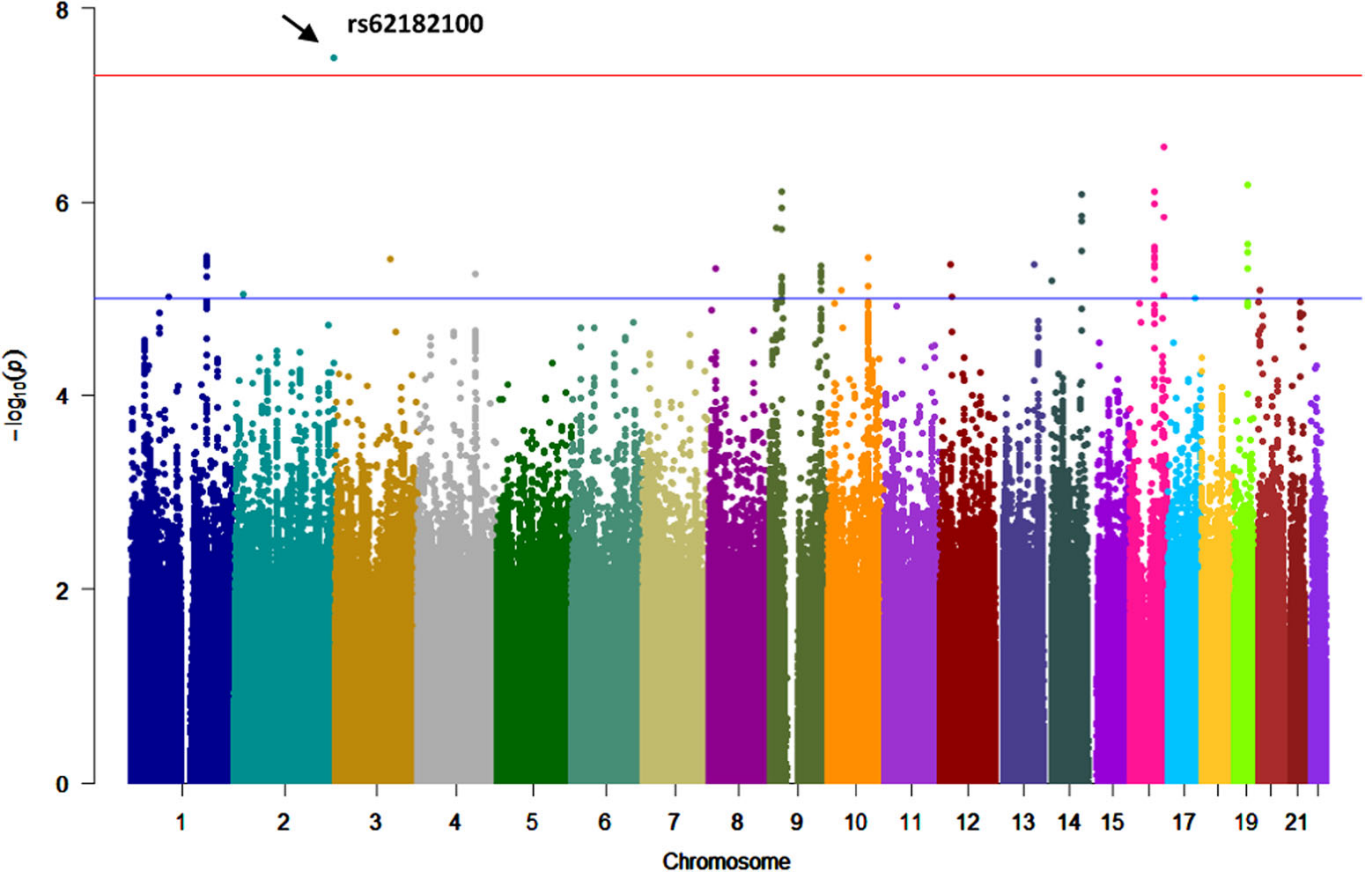
Manhattan plot depicting genome-wide significant loci associated with PCA-derived indices of IIRTV. The Manhattan plot depicts a genome-wide significant locus located on chromosome 2 for our measure of selective attention variability (ICV factor 2). Red line denotes a genome-wide significance threshold of 5 × 10^−8^, while the blue line represents a nominal significance threshold of 1 × 10^−5^ (note: −log10 *p* of the *p*-value of SNPs in the GWAS plotted along *y*-axis)

**Fig. 3:**
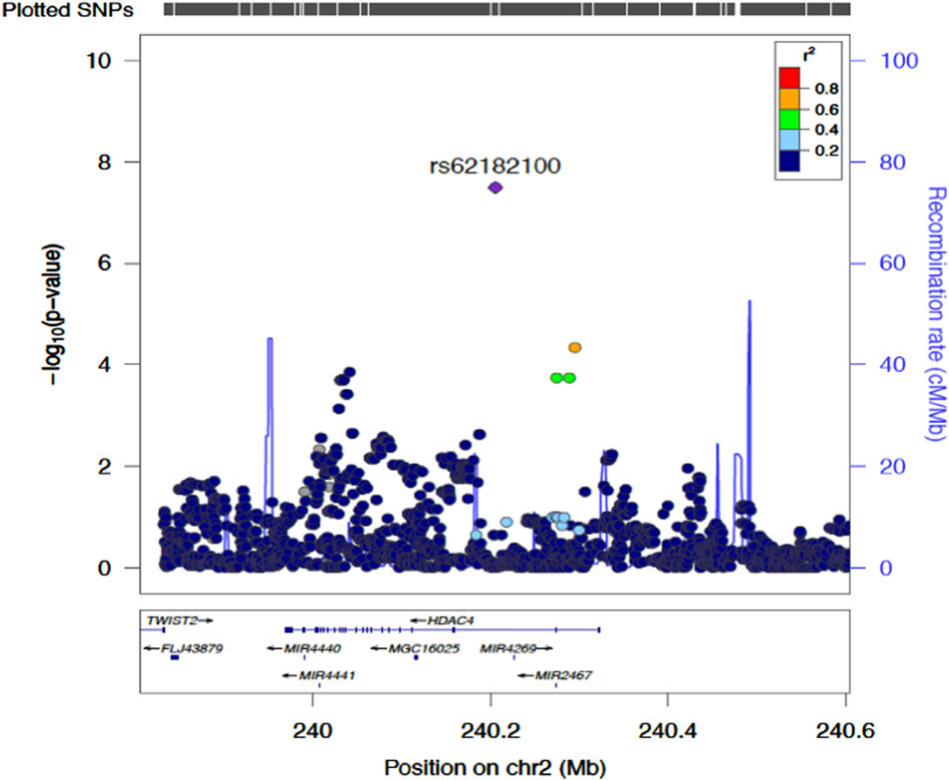
Regional association plot and recombination rates of the genome-wide significant locus (plotted in LocusZoom^36^) reveals numerous genes in and amongst the region. For our measure of selective attention variability (ICV factor 2), −log_10_ *p* of SNPs in the GWAS were plotted against their respective chromosomal locations on chromosome 2. The genome-wide significant SNP (rs62182100) is indicated by the diamond symbol, whereas circles the other SNPs located within the region. Estimated recombination rates (cM/Mb) are shown by the blue line. SNPs are colour-coded based on their pairwise *r*^2^ relative to the marker SNP. The genome-wide significant SNP is located in an intron of the gene *HDAC4*

For all cognitive tasks, stimuli were presented on an 85 Hz, 12 × 16-inch cathode ray tube monitor positioned at a viewing distance of 65 cm (stimulus visual angle 3.6° × 3.3°) away from the participant. Participants were instructed to align their visual gaze onto a centrally positioned fixation cross (+) and to make their responses using a standard computer keyboard. Five cognitive tasks were completed in counterbalanced order: (1) an Eriksen flanker task^25^, where participants provided either a ‘left’ or ‘right’ response to a target arrow located centrally on a computer screen, while discounting four flanking distractors (Fig. 1a); (2) a choice response-time task, which required participants to make rapid motor responses towards two different ‘go’ stimuli, represented by an X and O (Fig. 1b); (3) a stop-signal task^26,27^, in which participants were again required to make rapid motor responses to a go stimulus but this time asked to withhold their response when this stimulus was followed immediately by a stop signal (represented by a red square surrounding the go stimulus for approximately 25% of all trials); (4) a spatial competition task, which required participants to indicate the orientation of a target letter (an upright or inverter letter ‘T’), while ignoring competing distractors (Fig. 1c); (5) a Posner cuing task^28^, which required participants to select targets at cued (valid, invalid or neutral) locations from amongst rival distractors (Fig. 1d). Across all tasks, the mean RT (ms) and standard deviation (SD) of RT for correct responses was measured. As individuals may differ on response-time variability measures simply because they have different processing speeds, we also calculated the intra-individual coefficient of variation (ICV, SD_RT_/*M*_RT_), which provides a measure of response-time variability that controls for differences in the baseline speeds of processing^14^. Full details of the above tasks have previously been reported^22^.

### GWAS of IIRTV

A GWAS was performed to test for an association between our PCA-derived indices of IIRTV and individual genetic variants (imputed SNPs) using a linear regression model that assumed an additive model of genetic inheritance. Regression models were adjusted for age, sex, age^2^, age × sex, testing site, and the top two eigenvectors for the underlying population sub-structure. A genome-wide significance threshold of 5 × 10^−8^ was adopted to adjust for multiple comparisons. Linkage disequilibrium (LD) analysis was subsequently performed for our lead SNP using PLINK v1.9^29^, and were plotted using Haploview 4.1^30^

### Mediation analysis

In the current study, we tested a mediational model of an endophenotype, which assumes that the causal path from gene to disorder/trait passes through an endophenotype^31^. To assess the influence of IIRTV as an endophenotype of ADHD symptoms we utilised the Preacher and Hayes^32^ bootstrapping macro in SPSS to bootstrap the sampling distribution of the indirect effect, where the indirect effect corresponds to a reduction in the strength of the gene/ADHD symptom association that is attributed to IIRTV. Bootstrapping estimates were based on 100,000 bootstrap samples.

## Results

### Genotyping and imputation

A stringent quality control process was conducted on all task and genotypic data (see Supplementary Information). After quality control, the remaining sample comprised of 857 subjects (489 females and 368 males; *M*_age_ = 22.14 years, SD_age_ = 4.82 years).

### PCA of IIRTV

For the SD and ICV RT data derived for each of the five response time tasks, PCA was performed separately across each testing site (Melbourne and Brisbane). Given the intrinsic correlation between measures of SD and ICV RT data, the analyses and results are presented for ICV only (see Supplementary Information for analysis and results of SD RT data). For each PCA by site, the correlation matrices consisted of multiple coefficients above 0.3. Bartlett’s test of sphericity^33^ was significant and the Kaiser–Meyer–Oklin values surpassed the endorsed value of 0.6^34^, denoting an underlying latent structure in the response time data. For each testing site, two components in the PCA with eigenvalues over 1 were detected and retained. The retention of two components was further reinforced by a screeplot. For the measure of ICV, the two-component solution explained 56.582% and 56.579% of the variance for the Melbourne and Brisbane sites, respectively, with components 1 and 2 explaining 36.51% and 20.07% (Melbourne) and 37.2% and 19.38% (Brisbane) of the variance, respectively. Oblimin rotation^35^ was performed and revealed the presence of a simple structure, with our measure of ICV showing strong loadings and all task variables loading substantially to either one of two components (similarly for our measure of SD RT).

ICV from the Flanker, Go and Stop tasks loaded very strongly on component 1 (hereafter factor 1), while ICV from the Competition and Cueing tasks loaded very strongly on component 2 (hereafter factor 2; Supplementary Information Table 1 and 2). This factor structure confirmed a previous report from our group using a smaller but overlapping data set^22^.

Consideration of these loadings indicated that the first component was best characterised as a response selection variability factor; it predominantly contained response time tasks that required participants to select one response from competing response choices. The second component was best characterised as a selective attention variability factor; it comprised of response time tasks that necessitated participants to choose task-relevant from task-irrelevant stimuli, which did not map to a response alternative. Using the principal component regression method in SPSS version 24, estimated parameters from the PCA were used to define linear combinations of observed variables to generate factor scores that captured variations in response time in each individual. These PCA-derived indices of IIRTV were subsequently subjected to GWAS.

Both the response selective and selective attention variability factors related to self-reported ADHD symptoms (Diagnostic and Statistical Manual (DSM) IV Inattention; DSM IV Hyperactivity/Impulsivity; and ADHD Index) as assessed by the CAARS (see Supplementary Information; Table 3).

Both the response selective and selective attention variability factors related to self-reported ADHDsymptoms (Diagnostic and Statistical Manual (DSM) IV Inattention; DSM IV Hyperactivity/Impulsivity; and ADHD Index) as assessedby the CAARS (see Supplementary Information Table 3).

### GWAS of IIRTV

For our measure of selective attention variability (ICV factor 2), we identified one imputed SNP that attained genome-wide significance (see Figs. 2 and 3 for Manhattan and LocusZoom^36^ regional association plots, respectively): rs62182100 within the *HDAC4* gene (*P* = 3.24 × 10^−8^, *β* = 0.62). The imputation quality (*r*^2^) for rs62182100 is 0.51382—meeting the recommended INFO imputation quality score standard of 0.3 suggested to define sufficiently good imputation quality^37,38^, with a minor allele frequency of 0.05525. Comparable results were found for the measure of SD of RT (Supplementary Information: Figures 1 and 2 for SD factor 2 results). Manhattan plots for ICV factor 1 (and SD factor 1; without a genome-wide significant finding) are presented in Supplementary Information (Figure 3).

GWAS summary statistics for this study (https://figshare.com/s/60b39c8308e2a9986089) are open access and available under a Creative Commons Attribution-NonCommercial-ShareAlike 3.0 International License.

**Fig. 4:**
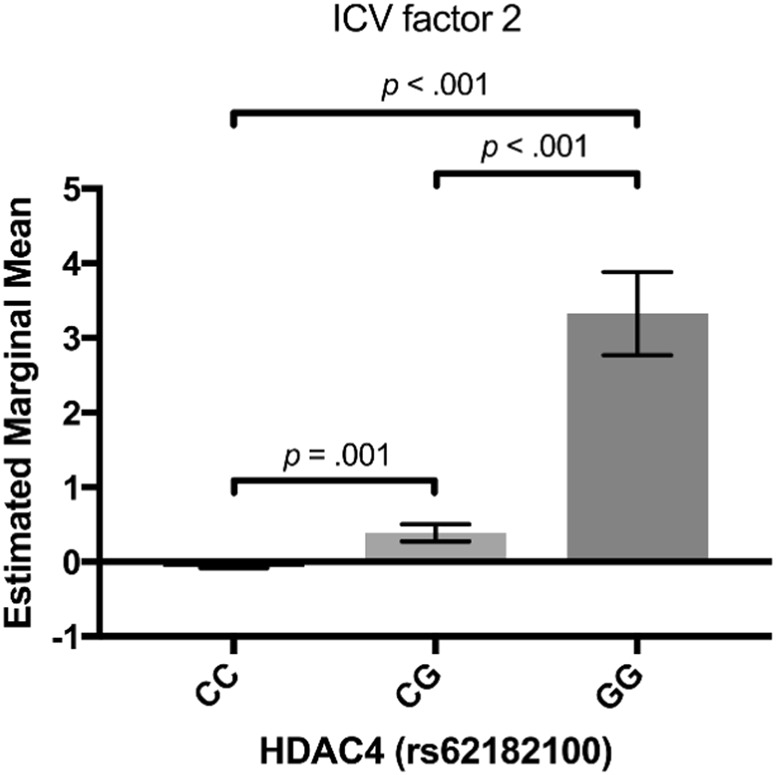
Genotype-by-phenotype plot for our measure of selective attention variability (ICV factor 2). The effect of the minor allele (genotype) (x-axis) on estimated marginal means (y-axis) for the genome-wide significant SNP (rs62182100). Errors bars represent SEM. Bonferroni-adjusted p-values are reported for pairwise comparisons (genotype) (*x*-axis) on estimated marginal means (*y*-axis) for the genome-wide significant SNP (rs62182100). Errors bars represent SEM. Bonferroni-adjusted *p*-values are reported for pairwise comparisons

Examination of the quantile-quantile plot for the distribution of *p*-values for ICV factor 2 (see Supplementary Information; Figure 4) indicated a close match to that expected for a null distribution except at the extreme tail of low *p*-values (similarly for SD factor 2; see Supplementary Information). This indicates more significant associations between genetic variants and IIRTV than expected by chance alone. The inflation factor of the test statistic for ICV factor 2 (*λ* = 1.01) revealed minimal inflation, suggesting little influence of population stratification or other systematic biases. LD analysis of our genome-wide significant SNP (rs62182100) identified three surrounding variants (those shown in orange and green in Fig. 3; rs62182145, rs62182153 and rs62182931), found to be in strong LD (*r*^2^ ≥ 0.5) with our lead SNP (see Supplementary Information; Figure 5).

To examine the addictive effect of the association between the *HDAC4* minor allele at rs62182100 and IIRTV we plotted the marginal means (after adjusting for age, sex, age^2^, age × sex, testing site, and the top two eigenvectors corresponding to the underlying population stratification). As can be seen from Fig. 4, an increase in IIRTV is demonstrated with increasing copies of the minor allele at rs62182100. This pattern was comparable for both factor 2 ICV and SD RT (see Supplementary Information; Figure 6). Associations for the three surrounding variants (rs62182145, rs62182153 and rs62182931) in strong LD with rs62182100 are additionally presented (see Supplementary Information; Table 4).

### Mediation analysis

We also assessed the influence of IIRTV as an endophenotype of ADHD symptomatology by evaluating whether any of the PCA-derived indices of IIRTV mediated an association between genetic variation in the genome-wide significant SNP (rs62182100) and measures of self-reported ADHD symptoms (ADHD Index, DSM IV Inattention, and DSM IV Hyperactivity/Impulsivity)^24^. The indirect effect of rs62182100 on the different self-reported ADHD symptom measures via factor 2 ICV are summarised in Table 1. As can be seen, the 95% bias-corrected bootstrap confidence intervals do not include zero, which indicates a statistically significant mediation effect. These data show that within a population of healthy young adults, IIRTV mediates the influence of the genetic variation in rs62182100 on self-reported ADHD symptoms.

**Table 1:** Point estimate effects (and 95% bias-corrected bootstrap confidence intervals) of the mediation of PCA-derived indices of IIRTV on the association between rs62182100 and self-reported ADHD symptoms (CAARS-S:L)^24^

Component	Conners’ Adult ADHD Rating Sub-scale		
	ADHD index	DSM IV Hyperactivity/impulsivity	DSM IV Inattention
ICV factor 2	0.802 [0.1958, 1.766]	0.808 [0.148, 1.938]	0.751 [0.091, 1.890]

## Discussion

Increased intra-individual response time variability is characteristically reported across a range of heritable neurological and psychiatric disorders. In disorders of attention, particularly ADHD, an increase in IIRTV is thought to be endophenotypic, indexing underlying genetic risk^12^. Here we have identified the first genetic locus associated with increased IIRTV through genome-wide analysis in a sample of healthy young adults. Further, we have also demonstrated that IIRTV mediates the relationship between the identified genetic variants present in the *HDAC4* gene and self-reported symptoms of ADHD.

This study reports for the first time a genetic association between DNA variation in the *HDAC4* gene and IIRTV. The *HDAC4* gene belongs to a class of histone deacetylase (HDAC) genes and encodes HDAC4^39^, which has been implicated in the indirect inhibition of DNA transcription^40^ through modulation of promoter activity^41^. HDACs play a pivotal role in the regulation of transcription factors by moderating their access to DNA^41^. Animal studies have previously implicated HDACs in the modulation of memory^42,43^, learning, and synaptic plasticity^44^, and pharmacological research has demonstrated that the administration of HDAC inhibitors lead to improvements in learning and memory^45^. Importantly, *HDAC4* is strongly expressed in the brain, being broadly expressed in the cortex and hippocampus^46^. Selective loss of *HDAC4* in the brain has been shown to result in impairments in spatial learning and long-term synaptic plasticity and spatial learning^44^. Although no study to date has directly implicated *HDAC4* in specific attentional processes, and given our lead SNP (rs62182100) lies within this gene, the relevant activity of this family of enzymes warrants further investigation within the context of attentional processes.

The results of this study are noteworthy for a number of reasons. First, to our knowledge it is the first study to employ a genome-wide approach to isolate the genetic substrates of IIRTV. Previous studies, including our own, have performed surveys of a limited number of candidate genes with mixed results^47–49^. Second, the observation that IIRTV mediates the relationship between DNA variation in the *HDAC4* gene and ADHD symptoms in healthy young adults, provides critical support for a mediational model of an endophenotype, under which genetic liability for a trait (i.e., ADHD symptoms) passes through the endophenotype^31^. Third, the fact that we identified our association with the “selective attention” and not “response selection” IIRTV factor is interesting. The cognitive tasks loading on the selective attention factor require participants to filter distractors and focus on task-relevant stimuli, which maps conceptually to subjective reports of “distractibility” in ADHD.

Nonetheless, there are a number of limitations to this study. First, our study is underpowered to detect reliable genome-wide discovery signals and lacked a replication sample. We suggest that a consortium approach to cognitive genetics—much like that used for imaging genetics (e.g., ENIGMA^50^)—will be needed to advance the field. One could argue that if IIRTV is an endophenotype for ADHD then genes linked to this trait should emerge as risk genes for ADHD more broadly. However, a simple look-up in the latest ADHD GWAS meta-analysis performed by the Psychiatric Genomics Consortium and Danish IPSYCH group^51^ found no evidence of association with either rs62182100 or the three SNPs in LD (rs62182145, rs62182153, and rs62182931) within *HDAC4*. Also, although one other study^52^ has recently reported cross-disorder evidence of association (*p* = 7.65 × 10^−6^) for rs3791556 located within the *HDAC4* gene and five major psychiatric disorders (including ADHD) the associated SNP (rs3791556) is not in LD with the SNP associated with IIRTV in this study. Interestingly, *HDAC4* has been implicated in functional genetic pathway analyses, with the class of histone methylation processes (GO Pathway IDs; GO:51568 and GO:16571) reportedly showing the strongest association of genetic expression related to the adult disorders (bipolar disorder, schizophrenia, and major depressive disorder)^53^. These observations suggest that our genome-wide findings should be interpreted with caution until replication samples become available. Indeed, we also note that we previously published a candidate gene association with IIRTV in a subset of the current sample (*N* = 402 subjects; 151 SNPs) and found an association with the SNPs in the ADRA2A gene and ICV factor 1 (response selection) (rs1800544 and rs602618)^22^. We do not, however find any consistent evidence for this association in the current genome-wide analysis in the sample of 857 young adults (*p*_corrected_ = 0.48 and 0.46 for rs1800544 and rs602618 variants, respectively).

In summary IIRTV captured by PCA-derived indices of response time variability were found to be associated with a genetic variation in the *HDAC4* gene and to mediate the relationship between this genetic variant of the *HDAC4* gene and self-reported ADHD symptoms. Although these data provide further support for the primacy of a response time variability endophenotype for ADHD symptoms, they require replication in larger cohorts.

## Electronic supplementary material

Supplementary Material
