# Supplementary material for: Genome-wide association study reveals novel genetic locus associated with intra-individual variability in response time
Source: Transl Psychiatry. 2018 Oct 4;8:207. doi: 10.1038/s41398-018-0262-z (PMC6172232; doi:10.1038/s41398-018-0262-z)
Supplement: Supplementary file 1 — Supplementary Material [file 41398_2018_262_MOESM1_ESM.docx]

Supplementary Information

*DNA Genotyping and Quality Control*

Genotyping of all samples was performed on the Illumina Infinium PsychArray-24 BeadChip platform^1^ at the PathWest Diagnostic Genomics Laboratory in Western Australia. The Illumina Psych-Chip is developed on a framework of 510,000 SNPs (265,000 SNPs markers found on the Infinium Core-24BeadChip and 245,000 markers from the Infinium Exome-24 BeadChip) and an additional ~50,000 SNPs associated with common psychiatric and neurodevelopmental disorders (developed in collaboration with the Psychiatric Genomics Consortium). Genotypes were called using GenomeStudio software which supports the Illumina arraybased genotyping applications and which converts microarray data into meaningful genotypes.

Genetic quality control was performed both at the individual and SNP level using PLINK version 1.9^2^ (www.cog-genomics.org/plink/1.9/) following a combination of stringent quality control protocols^3,4^.

*Per-individual Quality Control*

Before completing an assessment of SNP genotyping quality, individuals with low genotyping scores and with missing genetic data (⩾ 0.03), were identified, resulting in the exclusion of 23 individuals. Identity by descent analysis was subsequently performed to detect possible contamination of samples, duplications as well as unknown familial relationships (such as relatedness), resulting in the identification of 69 instances of relatedness which were excluded from further analysis. To assess and detect underlying population stratification, principal components analysis (PCA) was performed using the 1000 Genomes project data as reference panel (hg 19 build 37) and was implemented in PLINK version 1.9^2^. This resulted in the removal of 24 individuals. The top two eigenvectors for the underlying population (those with eigenvalues > 1) from the PCA analysis were further included as covariates in a regression framework (see section on principal components analysis within main text). Samples were also assessed for significant deviations in heterozygosity (denoted by greater than ±3 SDs from the sample mean), resulting in the exclusion of a further 47 individuals. An additional 39 individuals were excluded based on discordant sex information, as determined by observed X chromosome homozygosity^3^ (⩾ 0.02). Finally, only individuals for which cognitive data was collected were retained. In total, and after completion of individual quality control, a total of 439 individuals were excluded from all remaining analyses. The final sample comprised eight hundred and fifty-seven right-handed, healthy young adults (368 males and 489 females; M_age_ = 22.14 years, SD_age_ = 4.82 years).

*DNA imputation*

Imputation of genetic data was performed using MaCH version 1.0.16^5^ and Minimac version 2013.7.17^6^ as described in the ENIGMA imputation protocol (ENIGMA2 Genetics Support Team, 2012), using the Human Genome (hg19) reference genome, build 37. However, rather than using the 1000 Genome project containing genetic data for 41 million markers (of which ~23 million are monomorphic in Caucasians), we utilised a reference panel generated by ENIGMA2 containing 13,479,643 genetic variants observed more than once in European populations. Prior to imputation, SNPs deviating from Hardy–Weinberg equilibrium (*p* < 1 x 10^-5^) were removed (76 SNPs in total).

*Post Imputation Quality Control*

Following imputation, further data cleaning steps were implemented. Poorly imputed SNPs (*r*^2^ <0.3), were identified, resulting in the removal of 804,101 SNPs. During imputation, SNPs with a minor allele frequency (MAF ⩽ .01) were returned to the base dataset and therefore this filter was reapplied to the imputed dataset. SNPs allocated the same allele for both A1 and A2 or or which had ambiguous alleles assigned (A-T or G-C alleles) during imputation were also identified (132 in total) and removed. To minimize the likelihood of producing Type I errors a further 222,388 SNPs deviating from Hardy–Weinberg equilibrium (*p* < .001) were removed from the final dataset. Variants identified as either insertions or deletions (1,002,953 SNPs in total), were also excluded from the base dataset and remaining analyses. The application of these stringent quality control procedures resulted in the removal of 5,949,669 variants, leaving 7,529,974 SNPs (MAF frequency ⩾ 1%). However, a further 2,121,812 SNPs with a MAF of <.05 were removed from final GWAS analysis leaving 5,408,162 for association testing within our sample.

Table 1. Principal components analysis of IIRTV by testing site for ICV response time data derived from the five response time tasks.

|  | Component | Response time task measure | | | | | | |
| --- | --- | --- | --- | --- | --- | --- | --- | --- |
| Testing Site |  | Flanker | Go | Stop | Competition | | Cueing | |
| Melbourne | ICV factor 1 | .736 | .764 | .694 | |  | |  |
|  | ICV factor 2 |  |  |  | | .862 | | .573 |
| Brisbane | ICV factor 1 | .743 | .671 | .756 | |  | |  |
|  | ICV factor 2 |  |  |  | | .840 | | .675 |

Table 2. Principal components analysis of IIRTV by testing site for SD response time data derived from the five response time tasks.

|  | Component | Response time task measure | | | | | | |
| --- | --- | --- | --- | --- | --- | --- | --- | --- |
| Testing Site |  | Flanker | Go | Stop | Competition | | Cueing | |
| Melbourne | SD factor 1 | .747 | .700 | .806 | |  | |  |
|  | SD factor 2 |  |  |  | | .871 | | .684 |
| Brisbane | SD factor 1 | .712 | .729 | .780 | |  | |  |
|  | SD factor 2 |  |  |  | | .811 | | .842 |


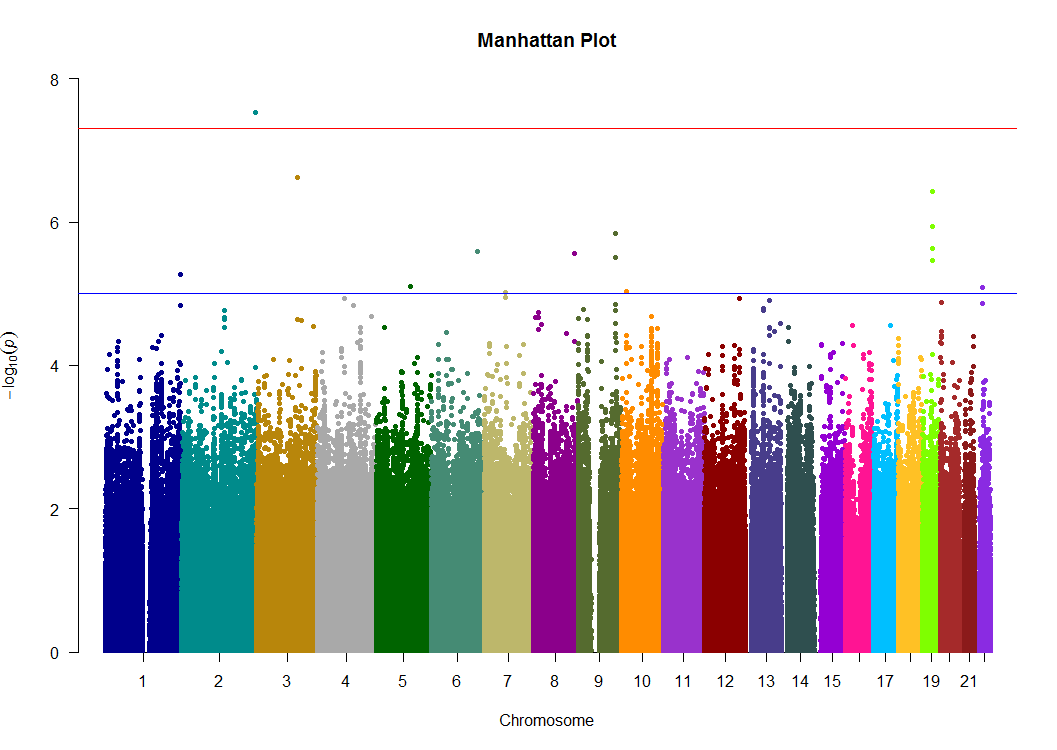


**rs62182100**

Figure 1. Manhattan plot depicting genome-wide significant loci associated with PCA-derived indices of IIRTV. The Manhattan plot depicts a genome-wide significant locus located on chromosome 2 for our measure of selective attention variability (SD factor 2), *P* = 2.93 × 10^−8^, β = .63 for SD factor 2. Red line denotes a genome-wide significance threshold of 5 x 10^-8^, while the blue line represents a nominal significance threshold of 1 x 10^-5^ (note: −log10 *P* of SNPs in the GWAS plotted along y-axis).


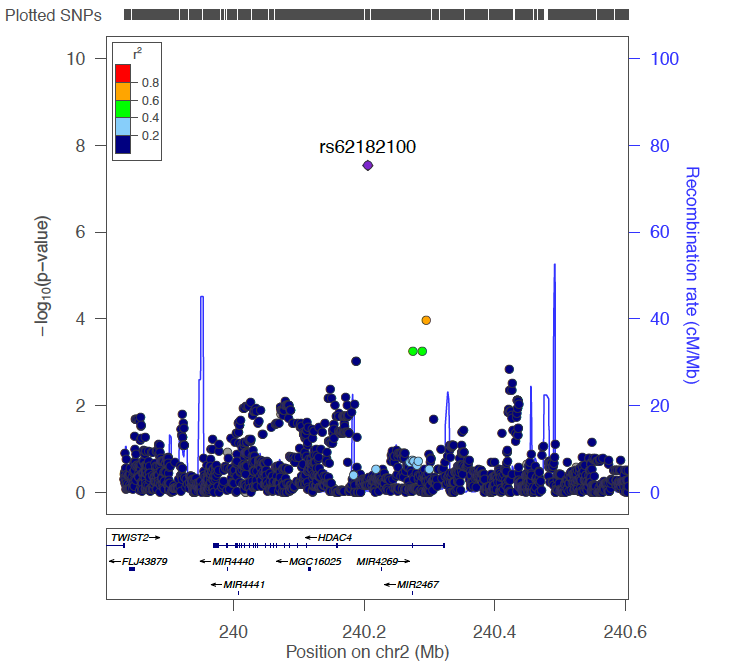


Figure 2. Regional association plot and recombination rates of the genome-wide significant locus (plotted in LocusZoom^7^) reveals numerous genes in and amongst the region. For our measure of selective attention variability (SD factor 2), −log_10_ *P* of SNPs in the GWAS were plotted against their respective chromosomal locations on chromosome 2. The genome-wide significant SNP (rs62182100) is indicated by the diamond symbol, whereas circles the other SNPs located within the region. Estimated recombination rates (cM/Mb) are shown by the blue line. SNPs are colour coded based on their pairwise *r*^2^ relative to the marker SNP.

**
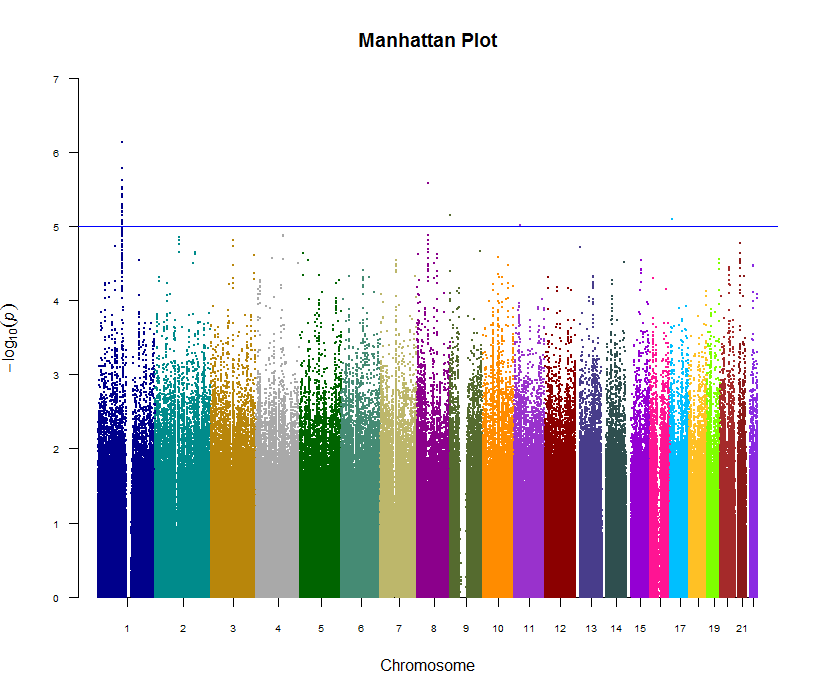
**

**A**


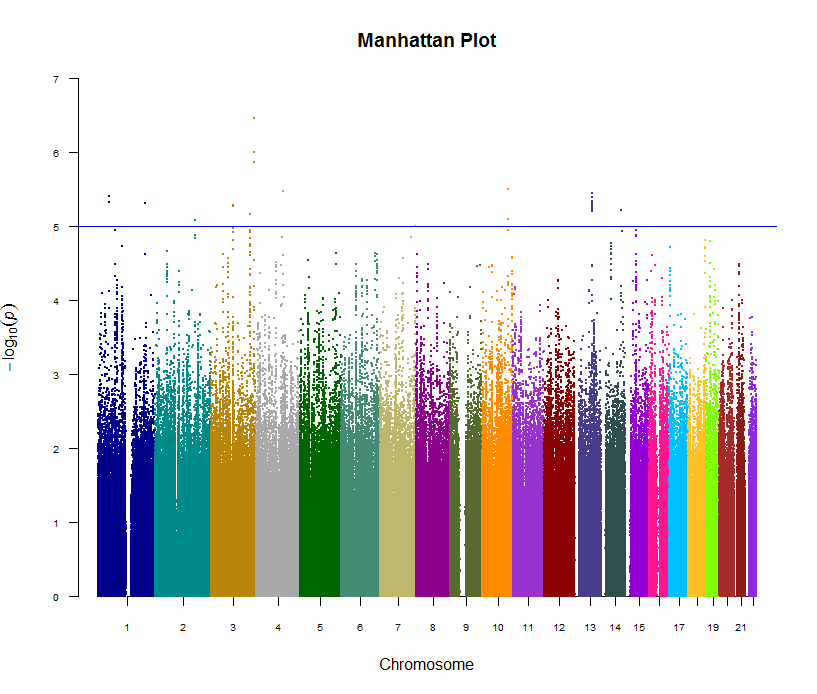


**B**

Figure 3. Manhattan plots depicting loci associated with PCA-derived indices of IIRTV. The Manhattan plot does not demonstrate any genome-wide significant loci for either factor 1 ICV (panel A) or SD (panel B). Blue line represents a nominal significance threshold of 1 x 10^-5^ (note: −log10 *P* of the *P-value* of SNPs in the GWAS plotted along y-axis).


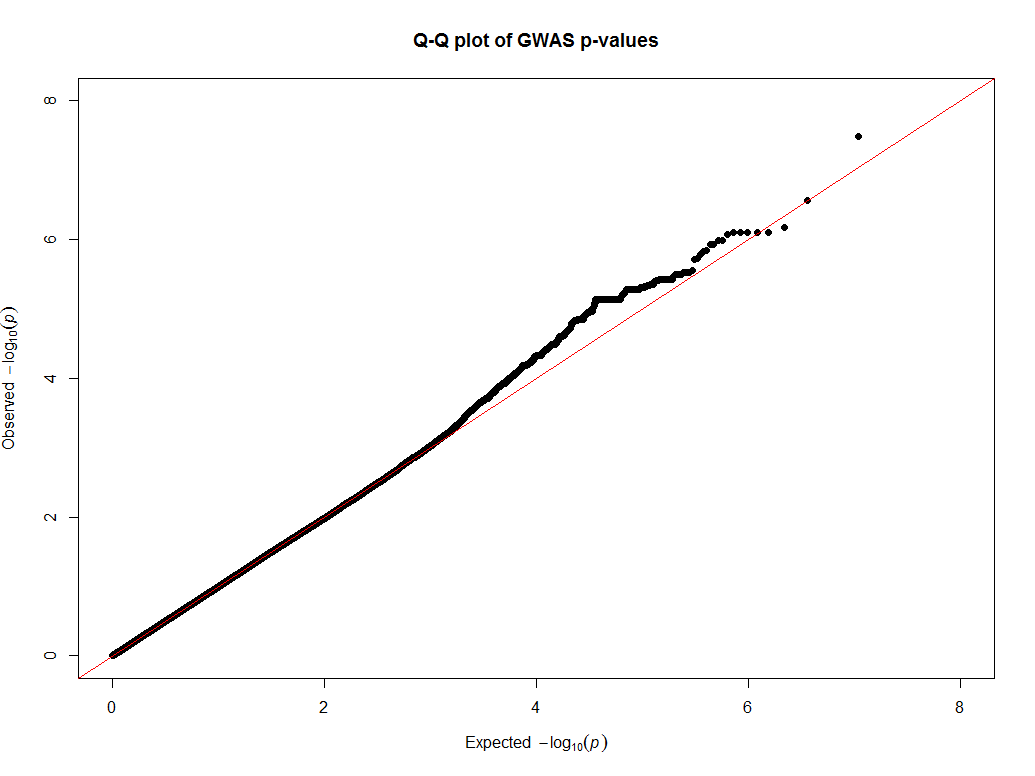

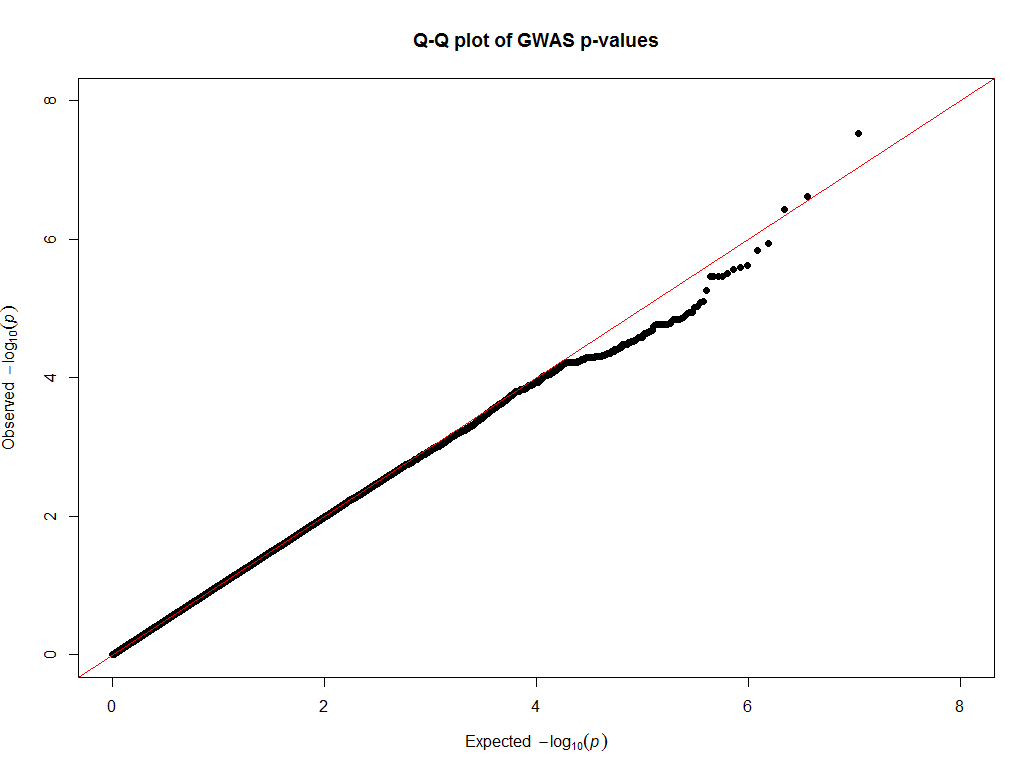


**B**

**A**

Figure 4. Quantile-Quantile (QQ) plot of the genome-wide associations studies for ICV factor 2 (panel A) and SD factor 2 (panel B). The QQ plot presents the distribution of expected *p*-values under a null distribution plotted against observed distribution. Both plots indicate slight inflation in the distribution of *p*-values, as indicated by λ = 1.01 and 0.99, for ICV factor 2 and SD factor 2, respectively.


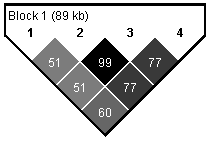


**rs62182100**

**rs62182145**

**rs62182153**

**rs62182931**


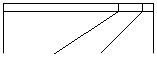


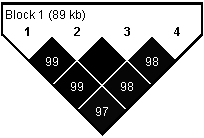


Figure 5. A diagram showing linkage disequilibrium (LD) relations (presented as *r*^2^) between the top GWAS-significant variant (rs62182100) and variants of the *HDAC4* gene (rs62182145, rs62182153 and rs62182931) plotted using Haploview 4.1^8^. The pairwise correlation coefficient (*r*^2^) multiplied by 100, indicating the degree of association between a pair of SNPs within the LD block is denoted numerically within each cell (top pyramid), along with the pairwise D’ values multiplied by 100 (lower pyramid).


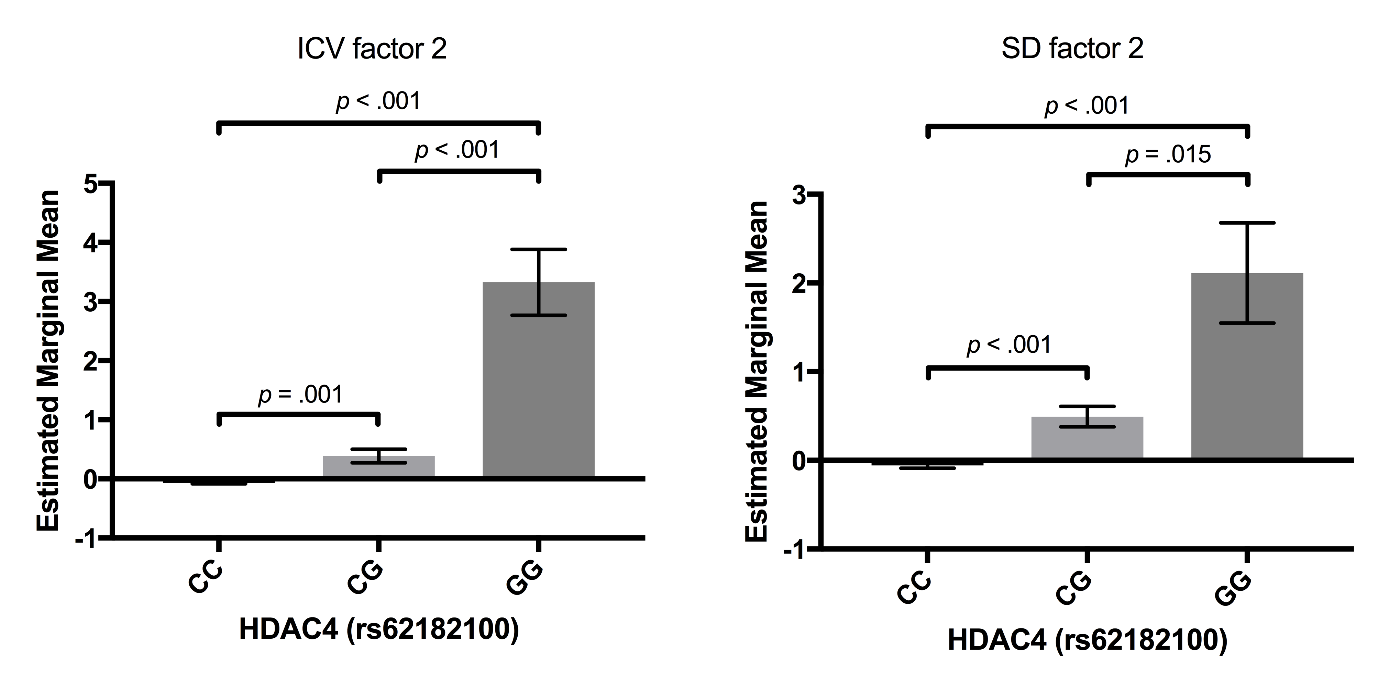


Figure 6. Genotype-by-phenotype plot for our measure of selective attention variability (SD factor 2) illustrating the effect of the minor allele (genotype) (x-axis) on estimated marginal means (y-axis) for the genome-wide significant SNP (rs62182100). Errors bars represent SEM. Bonferroni-adjusted *p*-values are reported for pair-wise comparisons. IIRTV differed significantly as a function of minor allele count: *F*(7,839) = 5.33, *R*^2^ = .043, *p* < .001.

Table 3. Partial correlations (*r*) between IIRTV (factor 1 and factor 2) and self-reported ADHD symptoms (ADHD Index; DSM IV Inattention; DSM IV Hyperactivity/Impulsivity) controlling for testing site (Melbourne / Brisbane), as assessed by the CAARS-S:L^24^,

| Component | Conners’ Adult ADHD Rating Sub-scale | | |
| --- | --- | --- | --- |
|  | ADHD index | DSM IV hyperactivity / impulsivity | DSM IV Inattention |
| ICV factor 1 | .136*** | .060 | .086* |
| ICV factor 2 | .114** | .130*** | .156*** |

Notes: **p* < .05. ***p* < .01. ****p* < .001 (two-tailed)

Table 4. Associations between LD variants of the *HDAC4* gene (rs62182145, rs62182153 and rs62182931) and IIRTV.

|  | **ICV factor 2** | | | | **SD factor 2** | | | |
| --- | --- | --- | --- | --- | --- | --- | --- | --- |
|  | *df* | *F* | *p* | *R^2^* | *df* | *F* | *p* | *R*^2^ |
| **rs62182145** | 7,838 | 3.67 | .001 | .03 | 7,839 | 3.25 | .002 | .026 |
| **rs62182153** | 7,838 | 3.67 | .001 | .03 | 7,839 | 3.25 | .002 | .026 |
| **rs62182931** | 7,838 | 4.06 | <.001 | .033 | 7,839 | 3.70 | .001 | .030 |

**References**

1. Infinium PsychArray-24 Kit | Psychiatric predisposition microarray [Internet]. Illumina.com. 2017 [cited 1 December 2017]. Available from: <https://www.illumina.com/products/by-type/microarray-kits/infinium-psycharray.html>
2. Chang C, Chow C, Tellier L, Vattikuti S, Purcell S, Lee J. Second-generation PLINK: rising to the challenge of larger and richer datasets. GigaScience. 2015;4(1).
3. Anderson C, Pettersson F, Clarke G, Cardon L, Morris A, Zondervan K. Data quality control in genetic case-control association studies. Nature Protocols. 2010;5(9):1564-1573.
4. Turner S, Armstrong L, Bradford Y, Carlson C, Crawford D, Crenshaw A et al. Quality Control Procedures for Genome-Wide Association Studies. Current Protocols in Human Genetics. 2011.
5. Li Y, Willer C, Ding J, Scheet P, Abecasis G. MaCH: using sequence and genotype data to estimate haplotypes and unobserved genotypes. Genetic Epidemiology. 2010;34(8):816-834.
6. Fuchsberger C, Abecasis G, Hinds D. minimac2: faster genotype imputation. Bioinformatics. 2014;31(5):782-784.
7. Pruim R, Welch R, Sanna S, Teslovich T, Chines P, Gliedt T et al. LocusZoom: regional visualization of genome-wide association scan results. Bioinformatics. 2010;26(18):2336-2337.
8. Barrett J, Fry B, Maller J, Daly M. Haploview: analysis and visualization of LD and haplotype maps. Bioinformatics. 2004;21(2):263-265.
